# The Combination of Particle Irradiation With the Hedgehog Inhibitor GANT61 Differently Modulates the Radiosensitivity and Migration of Cancer Cells Compared to X-Ray Irradiation

**DOI:** 10.3389/fonc.2019.00391

**Published:** 2019-05-14

**Authors:** Katrien Konings, Charlot Vandevoorde, Niels Belmans, Randy Vermeesen, Bjorn Baselet, Merel Van Walleghem, Ann Janssen, Sofie Isebaert, Sarah Baatout, Karin Haustermans, Marjan Moreels

**Affiliations:** ^1^Radiobiology Unit, Belgian Nuclear Research Center (SCK•CEN), Institute for Environment, Health and Safety, Mol, Belgium; ^2^Laboratory of Experimental Radiotherapy, Department of Oncology, KU Leuven, Leuven, Belgium; ^3^Radiation Biophysics Division, NRF iThemba LABS, Faure, South Africa; ^4^Laboratory of Morphology, Biomedical Research Institute (BIOMED), Hasselt University, Diepenbeek, Belgium; ^5^Department of Radiation Oncology, University Hospitals Leuven, Leuven, Belgium

**Keywords:** particle therapy, proton, carbon ion, radiosensitization, Hedgehog pathway, migration, gene expression, GANT61

## Abstract

Due to the advantages of charged particles compared to conventional radiotherapy, a vast increase is noted in the use of particle therapy in the clinic. These advantages include an improved dose deposition and increased biological effectiveness. Metastasis is still an important cause of mortality in cancer patients and evidence has shown that conventional radiotherapy can increase the formation of metastasizing cells. An important pathway involved in the process of metastasis is the Hedgehog (Hh) signaling pathway. Recent studies have demonstrated that activation of the Hh pathway, in response to X-rays, can lead to radioresistance and increased migratory, and invasive capabilities of cancer cells. Here, we investigated the effect of X-rays, protons, and carbon ions on cell survival, migration, and Hh pathway gene expression in prostate cancer (PC3) and medulloblastoma (DAOY) cell lines. In addition, the potential modulation of cell survival and migration by the Hh pathway inhibitor GANT61 was investigated. We found that in both cell lines, carbon ions were more effective in decreasing cell survival and migration as well as inducing more significant alterations in the Hh pathway genes compared to X-rays or protons. In addition, we show here for the first time that the Hh inhibitor GANT61 is able to sensitize DAOY medulloblastoma cells to particle radiation (proton and carbon ion) but not to conventional X-rays. This important finding demonstrates that the results of combination treatment strategies with X-ray radiotherapy cannot be automatically extrapolated to particle therapy and should be investigated separately. In conclusion, combining GANT61 with particle radiation could offer a benefit for specific cancer types with regard to cancer cell survival.

## Introduction

Over the past decades an increased use of charged particles in radiotherapy has been observed. This is due to the advantages that charged particles, such as protons and carbon ions, offer compared to conventional X-rays. More specifically, due to the physical properties of these particles, the tumor can be more precisely targeted while the healthy tissues receive a lower dose (1, 2). In addition, carbon ions are biologically more effective compared to X-rays. This is reflected by the relative biological effectiveness or RBE value for carbon ions which, on average, is around 2–3 (1, 3). Although it is assumed that protons have an RBE of ~1.1, and thus the same or a similar effect as X-rays, recent evidence shows that protons, at given conditions, are able to induce a different biological response in cancer cells compared to X-rays (4–7).

Many advances have been made to enhance the therapeutic index in conventional radiotherapy. Nevertheless, radioresistant tumors require high radiation doses and the level of dose that can be delivered to obtain local tumor control is limited by the tolerance of the surrounding healthy tissues to these high doses. One option to overcome this problem is to sensitize cancer cells to radiation, by means of chemical or molecular agents. This strategy has already been exploited in the context of conventional radiotherapy (8, 9). However, limited data exists about the combination of particle therapy with such agents. Moreover, carbon ion therapy can also be useful to treat radioresistant tumors due to the higher RBE of carbon ions.

Cancer patients diagnosed with metastasis, either before, during or after treatment, often have a worse prognosis. Several studies, both *in vitro* and *in vivo*, have observed an increased migratory potential of cancer cells after X-ray irradiation (10–13). On the other hand, particle irradiation has been found to mostly decrease their migratory potential (6, 14–18). Previous research of our group showed that carbon ions had a greater effect on global gene expression than X-rays and that genes involved in motility were more suppressed after carbon ions than after X-rays (19, 20).

It is known that several molecular pathways are involved in the process of metastasis, including the Hedgehog (Hh) signaling pathway. This pathway is important in early development as well as tissue repair and regeneration in adults and becomes activated after binding of the Hh ligands to the membrane receptor patched 1 (PTCH1). As a consequence, the inhibition by PTCH1 on Smoothened (SMO) is released and this leads, in the end, to the translocation of the Glioma-associated oncogene homolog (GLI) proteins to the nucleus and the regulation of the Hh target genes (21). These target genes are involved in angiogenesis, migration, invasion, cell cycle progression, and apoptosis. Aberrant signaling of the Hh pathway has been implicated in the development and progression of several tumor types (21, 22), cancer cell metastasis and resistance to X-ray radiation (23–25). Chen et al. observed that the addition of soluble sonic hedgehog (SHH) to hepatocellular cancer cells resulted in increased resistance to X-ray irradiation. In addition, exposing these cells to X-ray radiation was found to activate the Hh pathway (26, 27). Other *in vitro* and *in vivo* studies have also reported a link between radioresistance and the Hh pathway (28–30). A clinical study by Sims-Mourtada et al. found that esophageal cancer patients with an active Hh pathway could sustain the repopulation of esophageal cancer cells after chemo-irradiation (31). Overall, these studies clearly demonstrate the association between X-ray radiation and Hh pathway activation. Moreover, an active Hh pathway can lead to resistance to X-rays. To the best of our knowledge no data are available on the effect of particle irradiation on Hh pathway activation and the corresponding role in radioresistance.

Several different inhibitors of the Hh pathway have been developed, with SMO-inhibitors vismodegib and sonidegib being the only ones approved by the Food and Drug Administration. Unfortunately, resistance to SMO-inhibitors is often observed (32). Therefore, inhibiting the Hh pathway downstream of SMO might be more successful. One such downstream inhibitor is GANT61 (Gli-ANTagonist) which is an inhibitor of GLI1/2 (33). Combining radiotherapy with Hh inhibitors as a possible way to sensitize cancer cells to radiation, has already been investigated *in vitro* and *in vivo* in combination with X-rays (29, 34–36). In addition, several clinical papers have also reported the combination of vismodegib with X-ray radiotherapy in patients with basal cell carcinomas (37–41). However, research on the specific combination of GANT61 with X-ray irradiation is still limited and not available in combination with particle irradiation (30, 34, 42).

The aim of this study was to investigate the effect of X-ray, proton and carbon ion irradiation on cell survival, migration and Hh pathway gene expression. In addition, we explored the potential of the Hh inhibitor GANT61 as an effective modulator of radiosensitivity and migration of cancer cells for the different radiation types. Both prostate cancer and medulloblastoma cells were used in this *in vitro* study, because both tumor types are good indications for particle therapy (43) and the Hh pathway plays an important role in either the initiation or progression of these tumor types (44).

## Materials and Methods

### Cell Lines and Compound

Prostate cancer cells (PC3) and pediatric medulloblastoma cells (DAOY) were obtained from the American Type Culture Condition (ATCC, Molsheim Cedex, France). PC3 cells were cultured in minimal essential medium (Life technologies, Carlsbad, CA, USA) supplemented with 10% fetal bovine serum (FBS; Gibco, Life Technologies, Ghent, Belgium). DAOY cells were cultured in Eagle's Minimal Essential Medium (ATCC) supplemented with 10% FBS. All cell cultures were maintained in a humidified incubator (37°C and 5% CO_2_). Regular mycoplasma tests were performed on both cell lines. More information about the genetic background of both cell lines can be found on the website of the supplier (www.atcc.org).

For inhibition of the Hh pathway, the GLI1/2 inhibitor GANT61 was used at a concentration of 10 μM (Selleck Chemicals, Houston, TX, USA). Stock solutions were prepared by dissolving GANT61 in dimethyl sulfoxide (DMSO), then aliquoted and stored at −20°C. Control conditions were treated with the drug solvent. The drug was added 72 h before irradiation with either radiation type. During irradiation, the drug was still present in the medium until further processing of the cells was needed. After processing, GANT61 was removed.

### X-Ray Irradiation

Irradiation of the cells with X-rays was performed at the SCK•CEN facility (Mol, Belgium) using an Xstrahl 320 kV generator (250 kV, 12 mA, 3.8 mm Al, and 1.4 mm Cu). Cell culture flasks were horizontally irradiated with different X-rays doses [0, 0.25, 0.5, 2, 4, and 6 Gray (Gy)] at a calculated dose rate of 0.5 Gy/min.

### Proton Irradiation

Proton irradiation was performed at the iThemba LABS facility in South-Africa. Prior to the irradiation campaign, both cell lines were shipped from Belgium to South-Africa on dry-ice and cultured at iThemba LABS. Several days before the proton beam time was scheduled, cells were seeded in 12.5 cm^2^ flasks (Falcon, VWR; Leuven, Belgium). Just before irradiation, cell culture flasks were completely filled with medium and placed vertically in a Perspex phantom. The phantom consists of several Perspex plates with a cut-out for a culture flask to hold the cells at the specific depth, allowing a positioning accuracy of 0.1 mm. In order to irradiate the cells at a water equivalent depth (WED) of 85.0 mm that corresponds to a position near the middle of the SOBP, the required thickness of the total block of Perspex upstream of the measurement position was calculated by means of the numerical thick-target formalism proposed in Zhang and Newhauser (45) and the proton range and mass stopping power data given in Berger et al. (46). In addition, the wall of the cell culture flasks were accounted for in the WED calculations using a measured value for the density of the polystyrene material. Irradiations were performed with a 200 MeV proton beam, 10 cm field diameter, and a 50 mm SOBP with R50 range of 120 mm in water. The physical depth-dose profile of the proton beam was measured with a Markus™ ionization chamber (model 30-329) along the central axis of the Perspex phantom. These physical dose measurements were used to determine the output factor for the radiobiology experiments at the WED of 85.0 mm, which corresponds to an LET of 3.96 ± 0.20 keV/μm (47). The applied doses were 0.25, 0.5, 2, 4 and, 6 Gy. Due to the limited availability of the proton beam time, no samples for gene expression analysis, and migration assays could be obtained for the combination of proton irradiation with GANT61, for both cell lines.

### Carbon Ion Irradiation

Carbon ion beam time was granted by the iPAC committee of the Grand Accélérateur National d'Ions Lourds (GANIL; Caen, France). The cells were transported by car in a transportable incubator in completely filled flasks from Belgium to France. Afterwards the culture medium was replaced and the cells were placed overnight in a humidified incubator. Several days before irradiation the cells were seeded in 12.5 cm^2^ flasks (Falcon, VWR; Leuven, Belgium). Since irradiation was performed in a vertical position (perpendicular to the horizontal carbon ion beam), the culture flasks were completely filled with medium just before the start of the irradiation. Irradiation of the cells was performed with an initial carbon ion beam energy of 95 MeV/u (LET = 73 keV/μm) near the middle of the SOBP. The doses applied were 0, 0.25, 0.5, 1, 2, 3, and 4 Gy. Carbon ion dosimetry was performed as previously described (48).

### Colony Survival Assay

After irradiation of the cells in culture flasks, cells were trypsinized and seeded in triplicate in six-well plates at low densities. Fourteen days after seeding, cells were fixed with 6% glutaraldehyde and 0.5% crystal violet (Sigma). Colonies of more than 50 cells were counted with a ColCount colony counter (Oxford Optronix, Oxford, UK). To calculate the surviving fraction (SF), the following formula was used:

$$\begin{array}{lll} Plating\;efficiency\;\left(PE\right) & = & \frac{No.\;of\;colonies\;counted}{No.\;of\;colonies\;seeded}\;x\;100\%;\\ Surviving\;fraction\;\left(SF\right) & = & \frac{PE_{irradiated\;cells}}{PE_{Control}}\;x\;100\%. \end{array}$$

The survival curve was fitted to the LQ model using the following formula:

$$\begin{array}{l} SF=e^{-\left(\alpha D+\beta D^{2}\right)}, \end{array}$$

in this formula α and β are radiation sensitivity parameters, D is the irradiation dose. At least three independent experiments were performed with three replicates per experiment. Due to experimental limitations, we were not able to obtain a larger number of control samples (compared to irradiated samples). Therefore, it will not be possible to detect slight changes in the survival of cells at lower doses such as 0.5 Gy. The RBE was calculated at a 10% survival by dividing the dose of X-rays at SF_10_ by the corresponding dose of proton or carbon ions at SF_10_. In order to determine the effect of the drug the sensitizer enhancingratio (SER_10_) was calculated by taking the ratio of the doses to reach 10% survival for control cells over the treated cells.

### Gene Expression Analysis

RNA was isolated at 8 h and 24 h after irradiation using the Allprep DNA/RNA kit (Qiagen, Venlo, The Netherlands) according to the manufacturer's protocol. After measuring the RNA concentration with the DropSense 16 (Trinean, Ghent, Belgium), RNA was reverse transcribed to cDNA using the GoScript™ Reverse Transcription System (Promega, Leiden, The Netherlands). Finally, qPCR reactions were performed by the 7,500 Fast Real Time PCR system (Applied Biosystems, Foster City, CA, USA). Primers for the Hh pathway genes (*SHH, PTCH1, SMO, GLI1, GLI2, GLI3*, and *SUFU*) and target genes of the Hh pathway (*CYCLIND1, VEGFA, BCL-2, SNAIL*, and *MMP9*) can be found in Table 1. Specific target genes of the Hh pathway were selected based on their involvement in cell cycle regulation (CYCLIND1), apoptosis (BCL-2), or migration/invasion (VEGFA, SNAIL, and MMP-9).

**Table 1:** Primer sequences for target genes.

**Gene**	**Forward 5^**′**^-3^**′**^**	**Reverse 5^**′**^-3^**′**^**
GAPDH	CCATCTTCCAGGAGCGAG	TGAAGACGCCAGTGGAC
HPRT	TCAGGCAGTATAATCCAAAGATGGT	AGTCTGGCTTATATCCAACACTTCG
SHH	CCCGACATCATATTTAAGGATGAAGA	AAGCGTTCAACTTGTCCTTAC
PTCH1	AAACAGGTTACATGGATCAGATAATAG	CCCTTCCCAGAAGCAGT
SMO	ACCTATGCCTGGCACACTTC	GTGAGGACAAAGGGGAGTGA
GLI1	AATGCTGCCATGGATGCTAGA	GAGTATCAGTAGGTGGGAAGTCCATAT
GLI2	GCCCTCACCTCCATCAAT	TGTTCTGGTTGGTGTCACT
GLI3	GTGCTCCACTCGAACAGA	TCCAGGACTTTCATCCTCATTAGA
SUFU	CCATGAGTTTACAGGAACAGAT	GTGCCAAGCCCTGCATTA
CCND1	TGTAGTCACTTTATAAGTCATTG	CTTCAGCCATGAATAAGG
VEGFA	GCTACTGCCATCCAATCGAG	CTCTCCTATGTGCTGGCCTT
BCL-2	GGATGCCTTTGTGGAACTGT	AGCCTGCAGCTTTGTTTCAT
SNAIL	CCAATCGGAAGCCTAACTAC	AGAGTCCCAGATGAGCATTG
MMP9	GAACCAATCTCACCGACAGG	GCCACCCGAGTGTAACCATA

*GAPDH, glyceraldehyde 3-phosphate dehydrogenase; HPRT, Hypoxanthine-guanine phosphoribosyltransferase; SHH, sonic hedgehog; PTCH1, patched 1; SMO, smoothened; GLI, glioma-associated oncogene; SUFU, suppressor of fused; VEGFA, vascular endothelial growth factor; BCL-2, B-cell lymphoma 2; MMP9, matrix metallopeptidase*.

### Boyden Chamber Assay

To investigate the migratory potential of cancer cells after irradiation, the Boyden chamber assay (Neuroprobe, Gaithersburg, MD, USA) was used. Briefly, cells were seeded in serum-free medium in the upper wells of the Boyden chamber 24 h after irradiation. The lower wells were filled with medium containing 0.1% FBS. The upper wells were separated from the lower wells by means of a polycarbonate membrane with 8 μm pores (Neuroprobe). For PC3 cells the membrane was precoated with collagen I (Life Technologies, Carlsbad, CA, USA). After allowing the cells to migrate for 16 h, the cells were fixed with methanol and stained with 0.5% crystal violet. Images were captured with a Leica MZ12 microscope and the migrated cells were counted in five separate fields with Image J.

### Statistical Analysis

Statistical analysis was performed using GraphPad Prism 7 (GraphPad Software, Inc., La Jolla, USA). For the *in vitro* experiments, a one-way ANOVA with Tukey's multiple comparison test or a two-tailed student's *t*-test was performed. *P*-values ≤ 0.05 were considered statistically significant.

## Results

### Carbon Ion Irradiation Is More Effective in Cell Killing Compared to X-Ray or Proton Irradiation

**Figure 1:**
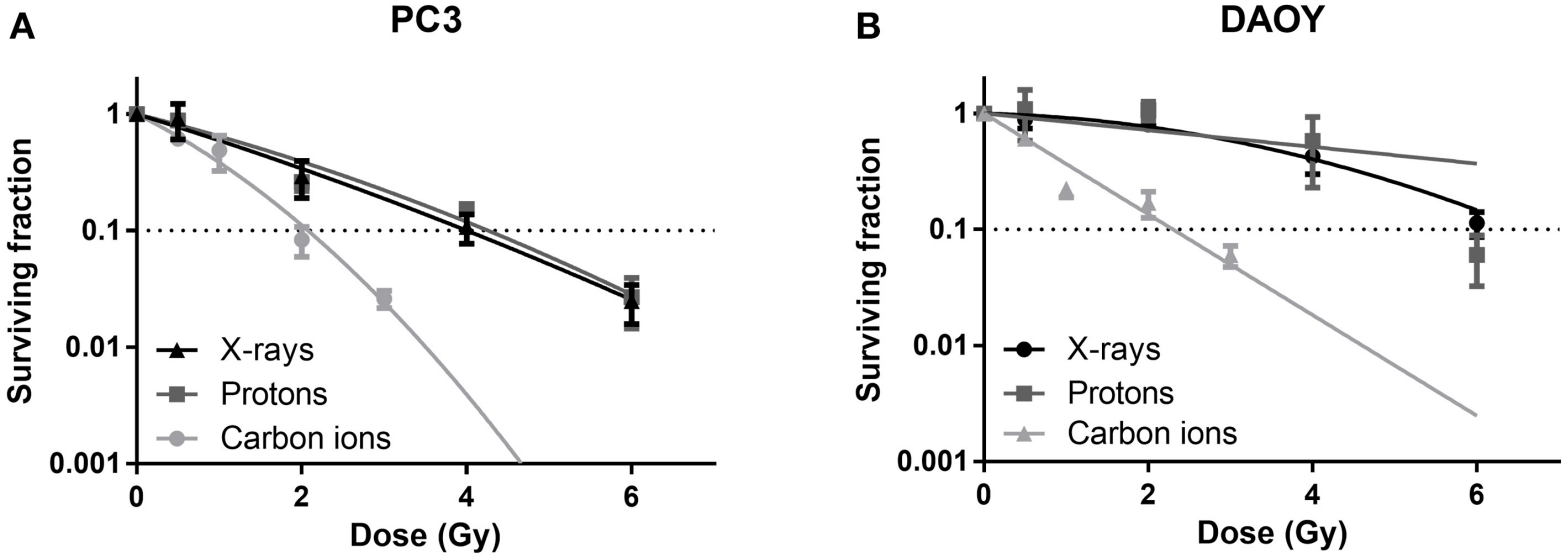
Colony survival assay of PC3 and DAOY cells irradiated with X-rays, protons or carbon ions. Surviving fraction of PC3 **(A)** and DAOY **(B)** cells after irradiation with different doses, calculated 14 days after start of colony survival. Means ± SEM of three independent experiments performed in triplicate.

All radiation types (X-rays, protons, and carbon ions) were able to induce a dose-dependent decrease in cancer cell survival (Figure 1). Proton irradiation induced a similar survival profile as X-rays, both in PC3 and DAOY cells. This was also reflected in the calculated RBE of protons at 10% survival (RBE_10_) which was 0.94 for PC3 cells and 1.06 for DAOY cells. In contrast, carbon ions were more effective in decreasing the survival of PC3 and DAOY cells compared to both X-rays and protons. The calculated RBE_10_ for PC3 cells was 1.93 and 2.57 for DAOY cells. More information about the radiation sensitivity parameters α and β of both cell lines can be found in Table 2.

**Table 2:** Radiation sensitivity parameters for PC3 and DAOY cell survival.

		**α**	**β**
PC3	X-rays	0.502	0.018
	Protons	0.408	0.031
	Carbon ions	0.810	0.144
DAOY	X-rays	0.039	0.047
	Protons	0.166	~ 0
	Carbon ions	~ 1	~ 0

*α and β are variables in the linear quadratic model which is as follows: SF = e-(αD+βD^2^)*.

### Different Radiation Types Induce Different Changes in Hedgehog Pathway and Target Genes

The investigated genes had a varied expression in response to the different radiation types (Table 3). In PC3 cells it is clear that carbon ions were able to induce more significant changes in the Hh pathway genes compared to protons and X-rays. Moreover, 8 h after carbon ion irradiation, all significant changes that are observed in the Hh pathway genes show an increased expression whereas at 24 h the majority of Hh pathway genes are significantly decreased. Significant decreases were observed at all doses of carbon ions for *SHH, PTCH1, SMO*, and *SUFU*. In contrast, very few significant changes in gene expression were observed after irradiation with X-rays and protons. Furthermore, 8 h after X-ray irradiation, most of the Hh pathway genes show a decreased expression whereas at 24 h this has shifted to an overall increased expression. At both time points after proton irradiation, the only significant change observed was at 24 h where *GLI3* showed a significant decreased expression for all the radiation doses. In contrast, for X-rays a significant increase in *GLI3* expression is observed at 24 h. The target genes of the Hh pathway also showed most significant changes after carbon ion irradiation. Specifically, at 24 h after carbon ion a persistent decrease in *VEGFA* could be observed. For X-rays and protons, an overall downregulation was observed at 8 h whereas an overall upregulation was observed at 24 h.

**Table 3:** Gene expression (qPCR) in PC3 cells after irradiation compared to control cells.

**PC3**	**X-rays**						**Protons**						**Carbon ions**					
	**8 h**			**24 h**			**8 h**			**24 h**			**8 h**			**24 h**		
	**0.5**	**2**	**4**	**0.5**	**2**	**4**	**0.5**	**2**	**4**	**0.5**	**2**	**4**	**0.5**	**2**	**4**	**0.5**	**2**	**4**
**Hh PATHWAY GENES**																		
SHH	0.98	0.66	0.06	0.77	0.12	0.15	0.96	0.37	0.62	0.77	0.92	0.98	0.66	0.78	0.82	< 0.0001 (^****^)	< 0.0001 (^****^)	< 0.0001 (^****^)
PTCH1	0.52	0.16	0.17	0.93	0.35	0.99	0.89	0.96	0.90	0.99	0.06	0.33	0.0002 (^***^)	0.45	0.08	< 0.0001 (^****^)	< 0.0001 (^****^)	< 0.0001 (^****^)
SMO	0.98	0.99	0.94	0.89	0.66	0.60	0.90	0.99	0.96	0.99	0.64	0.07	0.014 (^*^)	>0.99	0.64	0.007 (^**^)	0.07	0.04 (^*^)
GLI1	0.0004 (^***^)	0.001(^**^)	0.002(^**^)	0.99	0.21	0.04(^*^)	0.91	0.98	0.45	0.65	0.87	0.99	0.38	0.13	0.58	0.04 (^*^)	0.008 (^**^)	0.006 (^**^)
GLI2	0.71	0.98	0.86	0.99	0.48	0.43	0.58	0.89	0.80	>0.99	0.18	0.37	<0.0001 (^****^)	0.0002 (^***^)	0.007 (^**^)	0.25	0.24	0.002 (^**^)
GLI3	0.20	0.27	0.09	0.04 (^*^)	0.004 (^*^)	0.14	0.85	0.98	0.99	< 0.0001 (^****^)	< 0.0001 (^****^)	< 0.0001 (^****^)	0.0006 (^***^)	0.48	0.36	0.06	0.02 (^*^)	0.36
SUFU	0.99	0.99	0.39	0.86	0.93	0.99	0.98	0.56	0.27	0.99	0.37	0.33	0.41	0.99	0.97	0.003 (^**^)	< 0.0001 (^****^)	0.0001 (^***^)
**TARGETS GENES OF THE Hh PATHWAY**																		
CCND1	0.057	0.03 (^*^)	0.0008 (^***^)	0.99	0.17	0.01 (^*^)	0.61	0.64	0.20	0.88	0.12	0.13	<0.0001 (^****^)	0.18	0.006 (^**^)	0.98	0.99	0.002 (^**^)
BCL-2	0.79	0.13	0.07	> 0.99	0.95	0.93	>0.99	0.61	0.45	0.84	0.34	0.06	0.94	0.60	0.64	0.92	0.14	0.85
SNAIL	0.67	0.86	0.37	0.99	0.81	0.98	0.24	0.97	0.98	>0.99	0.97	0.31	<0.0001 (^****^)	0.49	0.32	0.94	0.99	0.24
VEGFA	0.72	0.85	0.94	0.83	0.83	0.29	0.96	0.14	0.19	0.75	0.39	0.007 (^**^)	0.64	0.02 (^*^)	0.10	< 0.0001 (^****^)	< 0.0001 (^****^)	< 0.0001 (^****^)
MMP9	> 0.99	0.49	0.88	0.93	0.93	0.72	0.99	0.61	0.34	0.78	>0.99	0.03 (^*^)	<0.0001 (^****^)	0.49	0.32	0.63	0.44	0.0004 (^***^)

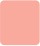
 non-significant decrease; 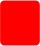 significant decrease;

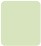
 non-significant increase; 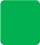 significant increase

values represent the p-values (compa to control, 0 Gy)

* p ≤ 0.05

** p ≤ 0.01;

*** p ≤ 0.001;

**** *p ≤ 0.0001*.

In DAOY cells, carbon ions induced much more significant changes in the expression of the investigated genes compared to X-rays and protons, for both 8 h and 24 h time points (Table 4). For the Hh pathway genes an overall decreased expression (non-significant) was observed both at 8 h and 24 h after X-rays. Moreover, a persistent decreasing trend could be observed for *SHH, PTCH1, SMO, GLI1, GLI2*. At 8 h after proton irradiation a decreased expression of the Hh pathway genes was observed. However, at 24 h most of these genes showed an increased expression. For carbon ions, only *SHH* demonstrated a persistent significant increased expression. The other genes of the Hh pathway showed a variable expression at 8 h after carbon ions whereas at 24 h most genes showed downregulation. All target genes of the Hh pathway, except for *CCND1*, showed a decreased expression 8 h after X-ray radiation. Only *SNAIL* and *MMP9* showed a persistent decreased expression after X-rays. *CCND1* showed a persistent increased expression after carbon ion radiation. Target genes of the Hh pathway mostly showed opposite responses at 8 h and 24 h after proton irradiation. For example, *CCND1* had an increased expression at 8 h and a decreased expression at 24 h after proton irradiation. In contrast, *BCL-2* and *VEGFA* showed an overall decreased expression at 8 h and an overall increased expression at 24 h after exposure to protons.

**Table 4:** Gene expression (qPCR) in DAOY cells after irradiation compared to control cells.

**DAOY**	**X-rays**						**Protons**						**Carbon ions**					
	**8 h**			**24 h**			**8 h**			**24 h**			**8 h**			**24 h**		
	**0.5**	**2**	**4**	**0.5**	**2**	**4**	**0.5**	**2**	**4**	**0.5**	**2**	**4**	**0.5**	**2**	**4**	**0.5**	**2**	**4**
**Hh PATHWAY GENES**																		
SHH	0.92	0.55	0.51	0.98	0.99	0.21	0.99	0.47	0.75	0.95	0.98	0.11	0.004 (^**^)	0.002 (^**^)	0.005 (^*^)	0.03 (^*^)	0.01 (^*^)	0.01 (^*^)
PTCH1	0.36	0.40	0.99	0.77	0.99	0.62	0.96	0.57	0.28	0.96	0.88	0.40	0.99	0.08	0.01 (^*^)	0.03 (^*^)	0.01 (^*^)	0.01 (^*^)
SMO	0.15	0.99	0.28	0.99	0.43	0.97	0.19	0.95	0.82	0.61	0.68	0.98	0.018 (^*^)	0.45	0.25	0.38	0.21	0.21
GLI1	0.64	0.18	0.65	>0.999	0.97	0.98	0.61	0.47	0.28	0.99	0.71	0.94	0.04 (^*^)	0.19	0.05	0.04 (^*^)	0.15	0.93
GLI2	0.15	0.28	0.78	0.62	0.99	0.61	0.99	0.59	0.46	>0.99	0.80	0.99	0.92	0.33	0.33	0.27	0.97	0.97
GLI3	0.16	0.97	0.28	0.89	0.67	0.97	0.04 (^*^)	0.04 (^*^)	0.03 (^*^)	0.75	0.59	0.93	0.03 (^*^)	0.79	0.66	0.96	0.31	0.30
**SUFU**	0.68	0.39	0.90	0.77	0.58	0.35	0.89	0.62	0.76	0.97	0.75	0.50	0.08	0.96	0.057	0.91	0.98	0.84
**TARGET GENES OF THE Hh PATHWAY**																		
CCND1	0.99	0.77	0.10	0.60	0.17	0.02 (^*^)	0.97	0.55	0.05	0.88	>0.99	0.0105 (^*^)	0.004 (^*^)	0.001 (^**^)	0.05	0.15	0.02 (^*^)	0.004 (^**^)
BCL-2	>0.99	0.25	0.12	0.94	0.95	0.79	0.77	0.009 (^**^)	0.002 (^**^)	0.04 (^*^)	0.99	0.86	0.99	0.16	0.39	0.03 (^*^)	0.15	>0.99
SNAIL	0.02 (^*^)	0.11	0.56	0.70	0.29	0.64	0.46	0.68	0.76	0.99	0.53	0.76	0.63	0.41	0.004 (^**^)	0.29	0.53	0.18
VEGFA	0.23	0.04 (^*^)	0.21	>0.99	0.99	0.48	0.69	0.11	0.03 (^*^)	0.99	0.85	0.53	0.85	0.71	0.89	0.03 (^*^)	0.054	0.01 (^*^)
MMP9	0.99	0.96	>0.99	0.52	0.06	0.98	0.93	0.98	0.30	0.33	0.72	0.04 (^*^)	0.28	0.49	0.01 (^*^)	0.25	0.82	0.46

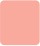
 non-significant decrease; 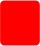 significant decrease;

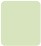
 non-significant increase; 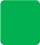 significant increase

values represent the p-values (compa to control, 0 Gy)

* p ≤ 0.05

** p ≤ 0.01;

*** p ≤ 0.001;

**** *p ≤ 0.0001*.

### Carbon Ion Radiation Suppresses Migration of PC3 and DAOY Cells More Than X-Rays or Protons

**Figure 2:**
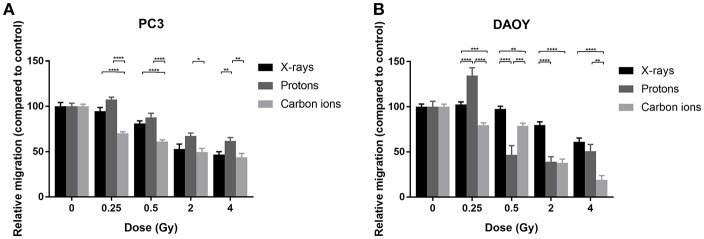
Migration of PC3 and DAOY cells after X-ray, proton, or carbon ion irradiation. The Boyden chamber assay was performed in order to assess the migratory potential of PC3 **(A)** and DAOY **(B)** cells. The migration assays was started 24 h after different doses of X-rays, protons, or carbon ions. Means ± SEM of three independent experiments performed in triplicate. ^*^p ≤ 0.05 vs. control cells; ^**^p ≤ 0.01; ^***^p ≤ 0.001; ^****^p ≤ 0.0001.

For both PC3 and DAOY cells, a general dose-dependent decrease in migration was observed for all types of radiation (Figure 2). In PC3 cells, protons were able to decrease the migration (except at 0.25 Gy), however the observed decrease was less pronounced than that of X-rays (Figure 2A). Carbon ions were significantly more effective in decreasing the migration of PC3 cells at low doses (0.25 and 0.5 Gy) compared to X-rays. In comparison to protons, carbon ions were overall more significantly effective in decreasing the migration of PC3 cells. In DAOY cells, protons were able to significantly decrease migration at a dose of 0.5 and 2 Gy in respect to similar X-ray doses (Figure 2B). In addition, carbon ions significantly decreased migration of DAOY cells at all doses compared to X-rays.

### GANT61 Sensitizes DAOY Medulloblastoma Cells to Particle Radiation

**Figure 3:**
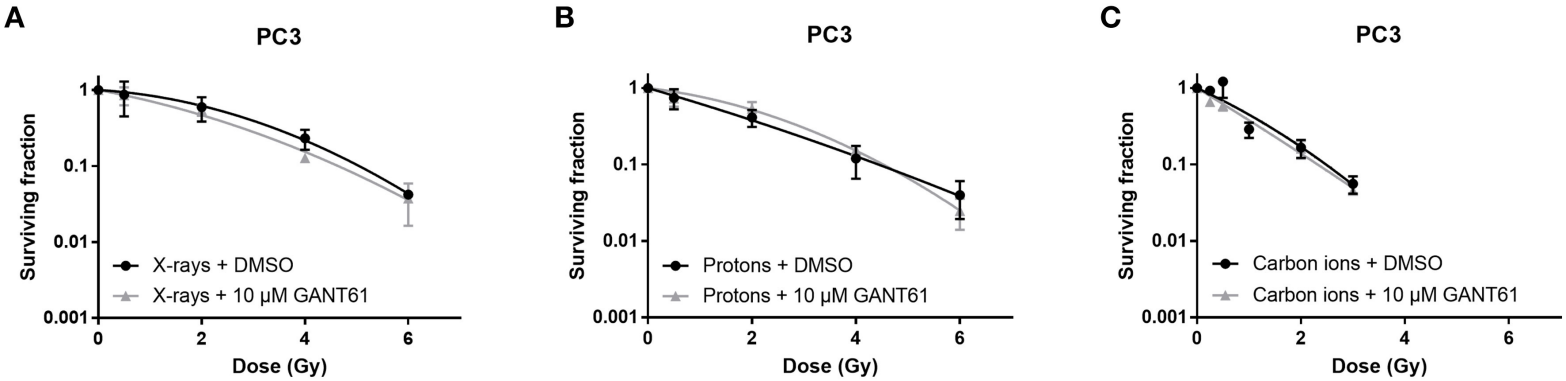
Effect of GANT61 in combination with radiation on the survival of PC3 cells. Colony survival curves of PC3 cells pre-treated for 72 h with GANT61 (10μM) and irradiated with different doses of X-rays **(A)**, protons **(B)**, or carbon ions **(C)**. Means ± SEM of three independent experiments performed in triplicate.

Survival of PC3 cells was not affected by the addition of GANT61 in combination with any of the radiation types (Figure 3). This was also reflected by a negligible sensitizer enhancing ratio at 10% survival (SER_10_) of 1.09, 0.98, and 1.07 for X-rays, protons, and carbon ions, respectively.

**Figure 4:**
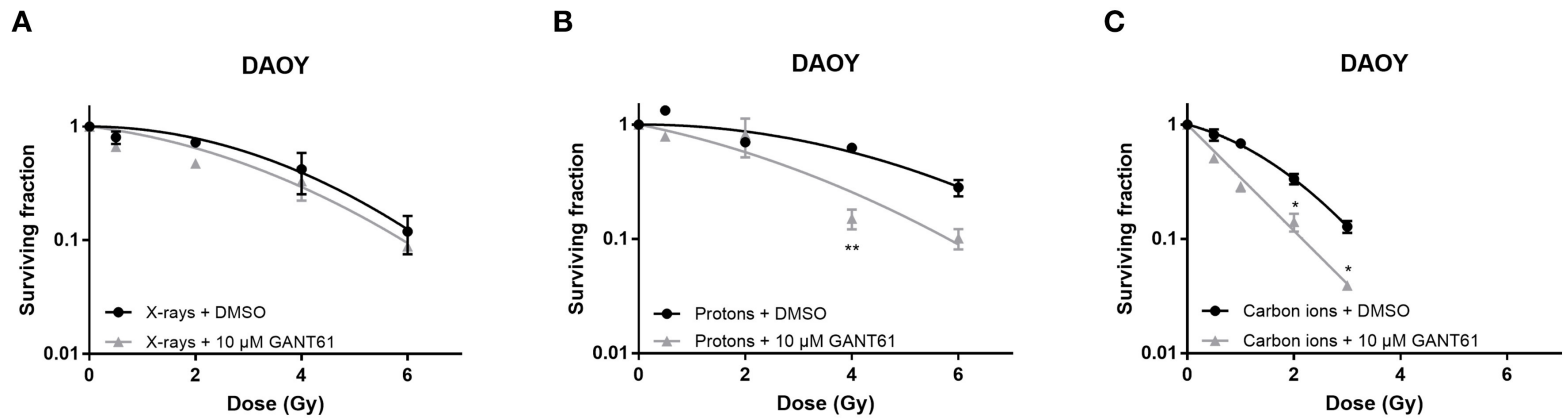
Effect of GANT61 in combination with radiation on the survival of DAOY cells. Colony survival curves of DAOY cells pre-treated for 72 h with GANT61 (10μM) and irradiated with different doses of X-rays **(A)**, protons **(B)**, or carbon ions **(C)**. Means ± SEM of three independent experiments performed in triplicate. ^*^p ≤ 0.05 vs. control cells; ^**^p ≤ 0.01.

In DAOY cells, GANT61 was able to sensitize the cells to proton and carbon ion radiation. Significant sensitization was observed at 4 Gy for protons and at 2 and 3 Gy for carbon ions. However, GANT61 was not able to sensitize DAOY cells to X-rays (Figure 4). The SER_10_ for X-rays, protons, and carbon ions was 1.07, 1.40, and 1.38, respectively.

### GANT61 Combined With Carbon Ion Radiation Are Able to Significantly Decrease Hh Pathway Gene Expression

The majority of Hh pathway genes showed a decreased expression after the combination of GANT61 with X-rays at 8 h post-irradiation (Table 5). The combination of GANT61 with carbon ions induced much more significant changes in the expression of the Hh pathway genes compared to the combination of GANT61 with X-rays. Moreover, 8 h after carbon ion irradiation, GANT61 was able to induce a significant decreased expression in the majority of the Hh pathway genes compared to DMSO treated cells. However, this was only persistent at 24 h after 0.5 Gy of carbon ions. For 2 Gy of carbon ions the opposite effect was observed after 24 h, with an increased expression in the majority of the Hh pathway genes. A significant decrease in *SUFU* gene expression was observed at 8 h after both X-rays and GANT61 and carbon ion and GANT61. The target genes of the Hh pathway showed an overall persistent decreased expression post-irradiation. In PC3 cells, the combination of GANT61 with either X-rays or carbon ions (Table 5) induced much more significant changes in gene expression as compared to irradiation alone (Table 3). In addition, opposite effects are observed after carbon ions compared to the combination of carbon ions with GANT61. Specifically for *SUFU*, which shows an increased expression at 8 h after carbon ions and a decreased expression at 24 h. In contrast, the combination of GANT61 and carbon ions significantly decreased the expression of *SUFU* at 8 h whereas an increase was observed at 24 h.

**Table 5:** Gene expression (qPCR) in PC3 cells after GANT61 treatment (10μm) of GANT61 in combination with radiation compared to DMSO treated cells.

**PC3**	**X-rays + GANT61**						**Carbon ions + GANT61**					
	**8 h**			**24 h**			**8 h**			**24 h**		
	**0.5**	**2**	**4**	**0.5**	**2**	**4**	**0.5**	**2**	**4**	**0.5**	**2**	**4**
**Hh PATHWAY GENES**												
SHH	0.11	0.65	0.01 (^*^)	0.07	0.16	0.03 (^*^)	0.^**^	^**^	^****^	^****^	^*^	
PTCH1	0.18	0.93	0.18	0.009 (^**^)	0.54	0.008 (^**^)	0.003 t^**^)	0.001 (^**^)	< 0.0001 (^****^)	0.0002 (^***^)	0.81	0.20
SMO	>0.99	0.29	0.95	0.01 (^*^)	0.89	0.006 (^**^)	0.16	0.04 (^*^)	< 0.0001 (^****^)	0.04 (^*^)	0.02 (^*^)	0.19
GLI1	< 0.0001 (^****^)	0.04 (^**^)	0.19	0.06	0.26	< 0.0001 (^****^)	0.08	0.38	0.003 (^**^)	0.12	< 0.0001 (^****^)	0.051
GLI2	0.25	< 0.0001 (^****^)	0.01 (^*^)	0.009	0.06	0.06	0.62	0.03 (^*^)	0.0005 (^***^)	0.006 (^**^)	0.02 (^*^)	0.36
GLI3	0.44	0.03 (^*^)	0.16	0.0008 (^***^)	0.93	0.0009 (^***^)	0.07	0.01 (^*^)	< 0.0001 (^****^)	0.006 (^**^)	0.02 (^*^)	0.36
SUFU	< 0.0001 (^****^)	< 0.0001 (^****^)	< 0.0001 (^****^)	0.03 (^*^)	0.78	0.79	0.002 (^**^)	0.003 (^**^)	< 0.0001 (^****^)	0.18	0.012 (^*^)	0.43
**TARGET GENES OF THE Hh PATHWAY**												
CCND1	0.002 (^**^)	< 0.0001 (^****^)	0.18	0.09	0.003 (^**^)	0.002 (^**^)	0.02 (^*^)	0.52	0.0009 (^***^)	0.00013 (^***^)	0.01 (^*^)	0.10
BCL-2	0.79	0.002 (^**^)	< 0.0001 (^****^)	0.53	0.007 (^**^)	0.0006 (^***^)	0.99	0.002 (^**^)	0.0003 (^***^)	< 0.0001 (^****^)	0.40	0.28
SNAIL	0.02 (^*^)	0.53	0.02 (^*^)	0.99	0.09	0.67	0.03 (^*^)	< 0.0001 (^****^)	0.0005 (^***^)	0.003 (^**^)	0.0009 (^***^)	0.31
VEGFA	0.91	0.0004 (^***^)	0.0006 (^***^)	0.002 (^**^)	0.35	< 0.0001 (^****^)	0.03 (^*^)	0.04 (^*^)	< 0.0001 (^****^)	0.10	0.04 (^*^)	0.0012 (^**^)
MMP9	0.65	0.78	0.98	0.42	0.84	0.58	0.05 (^*^)	0.003 (^**^)	0.007 (^**^)	<0.0001 (^****^)	0.01 (^*^)	0.0002 (^***^)

Gene expression is compared to DMSO treated cells.

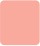
 non-significant decrease; 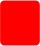 significant decrease;

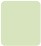
 non-significant increase; 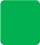 significant increase

values represent the p-values (compa to DMSO control)

* p ≤ 0.05

** p ≤ 0.01;

*** p ≤ 0.001;

**** *p ≤ 0.0001*.

In DAOY cells, the combination of GANT61 with X-rays had an overall increasing effect on the expression of the Hh pathway genes at 8 h and 24 h after treatment (Table 6). Furthermore, 24 h after treatment a significant decreased expression in *SMO* and *GLI1* was observed. For carbon ions, GANT61 was able to significantly increase the expression of most of the Hh pathway genes at 8 h post-irradiation, whereas at 24 h after carbon ion irradiation (2 and 4 Gy) all Hh pathway genes were significantly downregulated. The target genes of the Hh pathway showed an increased expression at 8 h post-irradiation (X-rays and carbon ions) whereas at 24 h post-irradiation the majority of genes were significantly downregulated. *BCL-2* showed a significant and persistent decreased expression at 8 h and 24 h after X-ray and GANT61 treatment. Comparing the effect of irradiation alone (Table 4) with the effect of GANT61 and irradiation demonstrated that the combination treatment induced much more significant changes compared to radiation alone. Moreover, X-rays alone downregulated the expression of *SNAIL* and *VEGFA* at 8 h post-irradiation whereas a significant increased expression was observed at 8 h after GANT61 and X-rays. For carbon ions we observed significant increases in *SHH, PTCH1, CCND1, BCL-2*, and *VEGFA* at 24 h post-irradiation. In contrast, the addition of GANT61 to carbon ions resulted in a significant downregulation of the aforementioned genes.

**Table 6:** Gene expression (qPCR) in DAOY cells after GANT61 treatment (10μM) in combination with radiation compared to DMSO treated cells.

**DAOY**	**X-rays + GANT61**						**Carbon ions + GANT61**					
	**8 h**			**24 h**			**8 h**			**24 h**		
	**0.5**	**2**	**4**	**0.5**	**2**	**4**	**0.5**	**2**	**4**	**0.5**	**2**	**4**
**Hh PATHWAY GENES**												
SHH	0.03 (^*^)	0.31	0.053	0.054	<0.0001 (^****^)	0.003 (^**^)	0.44	0.002 (^**^)	0.11	0.71	< 0.0001 (^****^)	< 0.0001 (^****^)
PTCH1	0.69	0.50	0.20	0.0105 (^*^)	0.09	0.0005 (^***^)	<0.0001 (^****^)	0.76	0.0002 (^***^)	0.06	< 0.0001 (^****^)	< 0.0001 (^****^)
SMO	0.44	0.06	0.03 (^*^)	0.006 (^**^)	0.02 (^*^)	< 0.0001 (^****^)	<0.0001 (^****^)	0.012 (^*^)	0.0002 (^***^)	0.0042 (^**^)	< 0.0001 (^****^)	< 0.0001 (^****^)
GLI1	< 0.0001 (^****^)	0.99	0.48	0.07	0.051	< 0.0001 (^****^)	0.014 (^*^)	0.72	< 0.0001 (^****^)	< 0.0001 (^****^)	< 0.0001 (^****^)	< 0.0001 (^****^)
GLI2	0.72	<0.0001 (^****^)	0.016 (^*^)	0.0109 (^*^)	<0.0001 (^****^)	0.004 (^**^)	0.77	0.015 (^*^)	0.0001 (^***^)	0.69	< 0.0001 (^****^)	< 0.0001 (^****^)
GLI3	0.32	0.0007 (^***^)	0.56	<0.0001 (^****^)	0.016 (^*^)	<0.0001 (^****^)	0.25	<0.0001 (^****^)	0.005 (^**^)	0.02 (^*^)	< 0.0001 (^****^)	< 0.0001 (^****^)
SUFU	0.09	< 0.0001 (^****^)	0.65	0.03 (^*^)	0.02 (^*^)	>0.99	0.059	<0.0001 (^****^)	0.0005 (^***^)	0.83	< 0.0001 (^****^)	< 0.0001 (^****^)
**TARGET GENES OF THE Hh PATHWAY**												
CCND1	0.03 (^*^)	<0.0001 (^****^)	0.16	0.10	0.12	0.14	0.005 (^**^)	0.83	0.24	<0.0001 (^****^)	< 0.0001 (^****^)	< 0.0001 (^****^)
BCL-2	0.07	< 0.0001 (^****^)	0.0006 (^***^)	0.008 (^**^)	0.03 (^*^)	0.03 (^*^)	0.18	0.04 (^*^)	<0.0001 (^****^)	< 0.0001 (^****^)	< 0.0001 (^****^)	0.0002 (^***^)
SNAIL	0.0003 (^***^)	<0.0001 (^****^)	0.80	0.009 (^**^)	0.12	0.41	<0.0001 (^****^)	<0.0001 (^****^)	0.0002 (^***^)	0.0003 (^***^-	< 0.0001 (^****^)	0.0004 (^***^)
VEGFA	0.0004 (^***^)	0.0013 (^**^)	0.0008 (^***^)	0.004 (^**^)	0.73	< 0.0001 (^****^)	<0.0001 (^****^)	0.02 (^*^)	<0.0001 (^****^)	0.20	< 0.0001 (^****^)	< 0.0001 (^****^)
MMP9	0.31	0.51	0.24	0.36	0.25	0.008 (^**^)	0.0014 (^**^)	0.26	0.46	0.27	< 0.0001 (^****^)	< 0.0001 (^****^)

Gene expression is compared to DMSO treated cells.

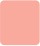
 non-significant decrease; 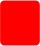 significant decrease;

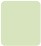
 non-significant increase; 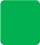 significant increase

values represent the p-values (compa to DMSO control)

* p ≤ 0.05

** p ≤ 0.01;

*** p ≤ 0.001;

**** *p ≤ 0.0001*.

### GANT61 Combined With Radiation Decreases Migration More Than Radiation Alone

**Figure 5:**
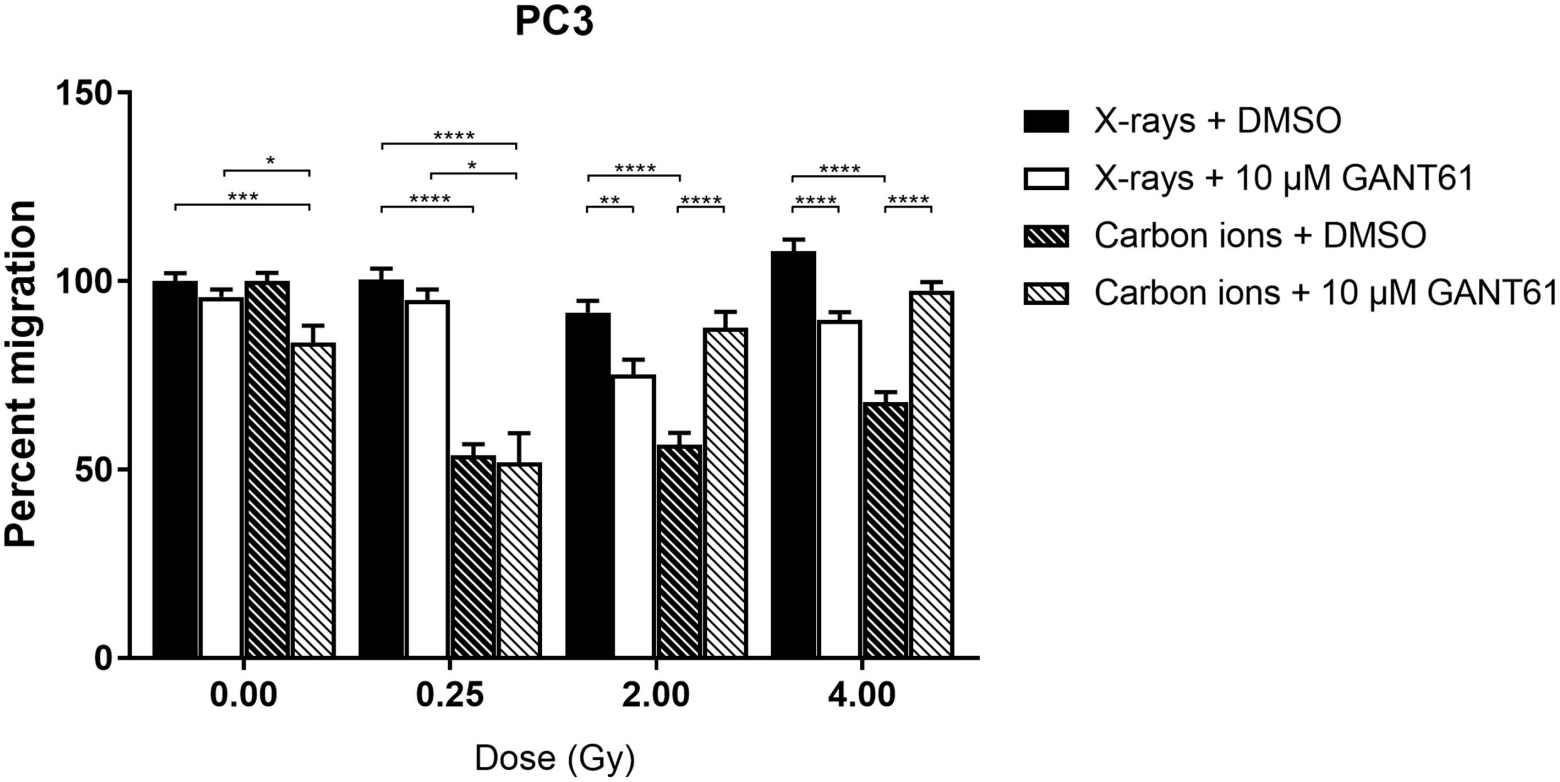
Migration of PC3 cells after the combination of GANT61 with irradiation. The Boyden chamber assay was performed in order to assess the migratory potential of PC3 cells after the combination of GANT61 (72 h pre-incubation; 10μM) with X-rays or with carbon ions. Means ± SEM of 2–3 independent experiments performed in triplicate. ^*^p ≤ 0.05 vs. control cells; ^**^p ≤ 0.01; ^***^p ≤ 0.001; ^****^p ≤ 0.0001.

Carbon ions were able to significantly decrease migration compared to X-rays in DMSO-treated PC3 cells at all doses except 0 Gy (Figure 5). The combination of X-rays and GANT61 was able to significantly decrease the migration of PC3 cells in comparison to DMSO treated controls at 2 and 4 Gy. In contrast, an increase in migration was observed after the combination of GANT61 with carbon ions compared to DSMO treated controls (Figure 5). However, at 0 and 0.25 Gy the combination of GANT61 with carbon ions was significantly superior at decreasing PC3 migration in comparison to GANT61 with X-rays.

**Figure 6:**
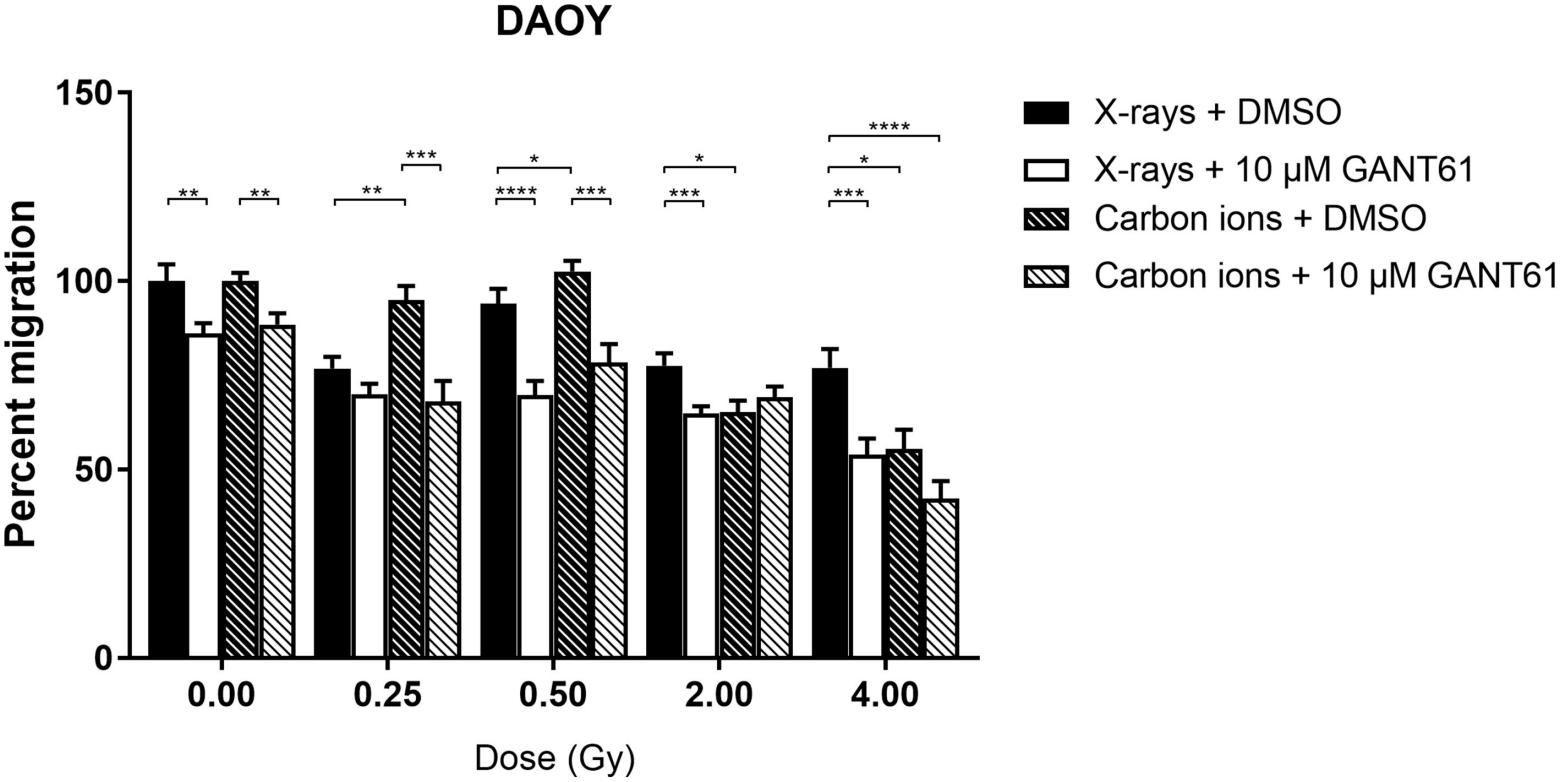
Migration of DAOY cells after the combination of GANT61 with irradiation. The Boyden chamber assay was performed in order to assess the migratory potential of DAOY cells after the combination of GANT61 (72 h pre-incubation; 10μM) with X-rays or with carbon ions. Means ± SEM of 2–3 independent experiments performed in triplicate. ^*^p ≤ 0.05 vs. control cells; ^**^p ≤ 0.01; ^***^p ≤ 0.001; ^****^p ≤ 0.0001.

Migration of DAOY cells was significantly more suppressed after carbon ion radiation compared to X-ray radiation at 2 and 4 Gy (Figure 6). The combination of X-rays with GANT61 was able to significantly decrease the migration in comparison to X-rays alone at all doses except at 0.25 Gy. The combination of GANT61 with carbon ions was only able to significantly decrease the migration of DAOY cells at lower doses (0.25, 0.5 Gy) in comparison to carbon ions alone.

## Discussion

With the increasing use of particle therapy in the clinic it is important to enhance our knowledge on the molecular mechanisms occurring in response to particle radiation as this might be different as compared to conventional X-ray therapy. Furthermore, combining radiotherapy with targeted therapies can sensitize cancer cells to radiation, thereby decreasing the radiation dose needed to obtain tumor control. Our previous studies showed that carbon ions are able to induce much more changes in global gene expression compared to X-rays in prostate cancer cells (20). In addition, carbon ions were found to be more effective than X-rays in decreasing the expression of genes involved in motility (19).

Previous studies showed that active Hh signaling can lead to resistance of cancer cells to X-ray radiation (28–30). Moreover, X-ray radiation can also activate the Hh pathway (26, 27). Finally, this pathway has also been linked to the migration and invasion of cancer cells. However, to the best of our knowledge, no investigations have been performed on the effect of particle radiation on the Hh pathway. Therefore, we investigated the effect of X-rays, proton, and carbon ion radiation on cell survival, migration, and Hh pathway gene expression. In addition, the potential modulating effect of the Hh inhibitor GANT61 on these endpoints was analyzed.

The effect of particle radiation on the survival of several cancer cells has already been investigated (49). However, there are no studies available that compare the effect of X-rays, protons, and carbon ions on cell survival in DAOY or PC3 cells in one single study. We observed that carbon ions were more effective in decreasing cell survival of both cell lines compared to either X-rays or protons. This is in line with other studies that observed similar findings in chordoma, colon carcinoma, and prostate cancer cells (19, 50). In addition, we observed that protons induced a similar survival curve as 250 kV X-rays. This was also reflected in the RBE_10_ of protons of 0.94 for PC3 cells and 1.06 for DAOY cells. The clinical RBE of protons is currently set to be ~1.1, however until today there is still much debate on whether this is correct or should be adjusted (51, 52). One of the arguments is that the RBE depends on several factors such as biological endpoint, linear energy transfer (LET), radiation dose, and tissue type. This is also clear from our RBE results, more specifically the difference in RBE between PC3 and DAOY cells, which reflects the variation between different cancer types.

We have investigated here for the first time the potential of the Hh inhibitor GANT61 to modulate the radiosensitivity of PC3 and DAOY cells in combination with proton and carbon ion irradiation. Our results indicate a radiosensitizing effect of GANT61 in combination with protons and carbon ions in DAOY cells, but not in combination with X-rays. In contrast, we could not observe a radiosensitizing effect of GANT61 in PC3 cells for any of the radiation types used in this study. So far there have only been a few studies investigating the combination of GANT61 with radiation, all of which have used X-ray radiation. Zhou et al. showed that GANT61 was able to sensitize renal cancer cells to X-rays (42). Another study by Gonnissen et al. investigated the effect of GANT61 in combination with X-rays in different prostate cancer cell lines. They observed a radiosensitizing effect in 22RV1 cells, but not in the DUI145 or PC3 cells (30, 34). This is in accordance with our results for PC3 cells where we also did not observe a radiosensitizing effect of GANT61 in combination with X-rays. GANT61 has been shown to induce sensitivity to X-ray radiation in a p53 dependent manner. Therefore, wild-type (WT) p53 cell lines will be sensitized to X-ray irradiation by GANT61, whereas cell lines with mutated p53 will not show any sensitization to X-ray irradiation (34). Both PC3 and DAOY cells have mutated p53 and this could explain why both cell lines do not show sensitization to X-ray radiation (53). On the other hand, it has been shown that particle radiation can induce radiosensitization in a p53 independent manner (54). This could explain the GANT61-induced sensitivity to proton and carbon ion radiation in DAOY cells. However, this could not be observed in the PC3 cells. One potential explanation might be that the basal Hh pathway activity is relatively higher in DAOY then in PC3 cells (data not shown). Since in PC3 cells only very low levels GLI1, a marker for Hh pathway activity, were observed, we could expect that Hh inhibition would not affect the cells much. In addition, gene expression at 24 h after the combination of carbon ion radiation + GANT61 showed significant decreases for the Hh pathway genes and its target genes in DAOY cells (2 and 4 Gy), whereas such a strong significant decrease was not observed in PC3 cells after the combination of carbon ion + GANT61 (except for at a 0.5 Gy dose). Another possible explanation could be that other mechanisms play a role in the response to the combination treatment. Moreover, GANT61 influences other target genes or processes, which we did not investigate here but that could have an effect and might explain the differences observed between both cell lines. Specifically, GANT61 targets not only cell cycle progression, migration, or apoptosis (as we have investigated the expression of some of the genes involved in these processes), but also DNA damage repair, autophagy, inflammatory response, limitless replicative potential, and CSC's are targeted by GANT61 (33).

In this study we showed that X-rays, protons, and carbon ions each induce their own expression profile of the Hh pathway and some selected Hh target genes. Moreover, we observed that, both in PC3 and DAOY cells, carbon ions were more effective in significantly altering the expression of the Hh pathway genes and target genes in comparison to X-rays or protons. This is in line with several microarray studies that investigated gene expression after X-ray and carbon ion irradiation. In these studies carbon ions were able to induce much more significant alterations in global gene expression compared to X-rays (20, 55, 56). Microarray studies comparing protons with X-rays are very scarce. One study by Girdhani et al. demonstrated that protons induced a dose-dependent downregulation in *VEGF, IL-6, IL-8*, and *HIF1A* expression in a panel of different cell types whereas X-rays showed a dose-dependent upregulation of the expression of these genes (4, 57). Next to the radiation-type dependent response of the Hh pathway gene expression, we also observed a cell-type dependent expression of the investigated genes. For example, at 24 h after X-ray irradiation an overall increasing trend is observed in the Hh pathway gene expression in PC3 cells whereas in DAOY cells the majority of Hh pathway genes show a downregulated expression. The opposite is observed 24 h after carbon ion radiation, which is most prominent for *SHH* and *PTCH1* which both are significantly downregulated in PC3 cells, whereas they are significantly upregulated in DAOY cells. For PC3, *VEGFA* was downregulated at 8 h after proton radiation whereas an upregulation was observed 24 h after protons and 8 h and 24 h after X-rays. These results are in line with a study by Girdhani et al. where X-rays induced an increased expression of *VEGFA*, whereas protons decreased the expression of *VEGFA* (4). This is in contrast to what we observed in DAOY cells, where *VEGFA* showed an overall decreased expression at 8 h after X-rays and protons whereas an overall increased expression was observed at 24 h. However, it should be noted that in the study by Girdhani et al. only the time point of 6 h post-irradiation was investigated. The increased expression of *CCND1* observed in PC3 and DAOY cells at 8 h after X-ray or carbon ion exposure is similar to what Fushimi and colleagues observed (58). In this study, an oral squamous cell carcinoma cell line was irradiated with either X-rays, carbon ions, or neon ions. They reported that both X-rays and carbon ions upregulated the expression of *CCND1* with carbon ions inducing a higher upregulation than X-rays. A study by Chun et al. observed a downregulation of *MMP9, SNAIL1*, and *VEGFA* after proton irradiation in liver cancer cells (59). This is in line with what we observed 8 h after proton radiation in PC3 cells (except for *SNAIL*) and in DAOY cells (except for *MMP9*). Again it should be noted that this study investigated gene expression at 6 h whereas we investigated at 8 h post-irradiation.

The combination of GANT61 with carbon ions was able to suppress even more the downregulation in the Hh pathway genes in PC3 and DAOY cells compared to DMSO treated controls. This decreased expression was more pronounced after carbon ions than after X-rays. At 24 h after carbon ion radiation a significant downregulation in Hh pathway gene expression was observed in DAOY cells. This could explain the radiosensitizing effect of GANT61 that we observed in response to carbon ions. In contrast to the PC3 cells, where the Hh pathway genes shows less repression at 24 h after carbon ions and no radiosensitizing effect was observed. We also observed cell-type dependent changes in gene expression after the combination treatment. Moreover, PC3 cells showed a decreased expression at 8 h after GANT61 and X-ray exposure, whereas DAOY cells showed an increased expression at 8 h and 24 h after X-ray and GANT61 exposure. The addition of GANT61 to either X-rays or carbon ions results in a significant downregulation of *BCL-2* in almost all investigated doses in both PC3 and DAOY cells. Since BCL-2 is known to promote cell survival, this could indicate that the addition of GANT61 to radiation affects cell survival even more than radiation alone. As previously mentioned, we were not able to obtain samples for gene expression after proton radiation and GANT61 treatment. It would therefore be interesting to gather this data in the future to see whether a similar effect is seen as with carbon ions and if this could be linked to the radiosensitization observed for protons.

Contradictory results exist concerning the effect of X-ray radiation on the migration of cancer cells, with some studies showing a decrease in migration while others show an increase in migration (10). In contrast, it is believed that carbon ions are more effective in decreasing the migration and invasion of cancer cells (14, 60). Our results show a dose-dependent decrease in migration after X-rays, protons as well as carbon ions, with carbon ions inducing a more pronounced decrease compared to X-rays and protons in both cell lines. This is in line with previous studies showing a decrease in migratory potential of DAOY cells after X-ray and carbon ion radiation (61). However, there are also some studies demonstrating an increased migration of DAOY cells in response to X-rays (62, 63). Moreover, we observed an increased migration of DAOY cells after a 0.5 Gy dose of protons. There have been some papers that observed an increased migration in response to proton irradiation, however this was at much higher doses (3, 4, and 8 Gy) (6, 64). For X-ray irradiation it is known that sublethal doses can increase the migration of cells, due to increased MMP activity and changes in BCL-2 family protein expression toward an apoptosis-resistant phenotype (11, 65). In line with these findings, it could also be that sublethal doses of proton irradiation might enhance the migration of cells. It would therefore be interesting to also investigate the expression of the migration related genes or MMP activity at lower proton doses (0.1–0.25 Gy).

No studies have been performed to investigate the effect of protons on DAOY cells or the effect of X-rays, protons, or carbon ions on PC3 cells. Based on our results, the migratory potential of DAOY cells appeared to be more affected than PC3 cells by irradiation.

The combination of GANT61 and radiation exposure on the migration of cancer cells has not been investigated so far. We show here for the first time that the combination of GANT61 with X-rays in PC3 and DAOY cells, is able to decrease migration more effectively than X-rays alone. We also investigated Hh target genes involved in cell migration (*SNAIL, VEGFA*, and *MMP9*) and found that both *SNAIL* and *VEGF* expression was decreased 24 h after the combination of X-ray and GANT61 exposure. This downregulation could possibly explain the significant decrease in migration of PC3 cells treated with X-rays and GANT61 compared to DMSO treated cells. In contrast, the combination of carbon ions and GANT61 increases the migration of PC3 cells at 2 and 4 Gy, which could be linked to the increased expression of *VEGFA* (2 Gy) and *MMP9* (2 and 4 Gy) at 24 h after GANT61 and carbon ion irradiation. In contrast, *SNAIL* shows a decreased expression at 2 and 4 Gy 24 h after the combination treatment. The combination of GANT61 with carbon ions was only able to significantly decrease the migration of DAOY cells for carbon ion doses up to 0.5 Gy compared to DMSO treated controls. For DAOY cells, *SNAIL, VEGF*, and *MMP9* expression was decreased 24 h after the combination of X-rays and GANT61 as well as the combination of carbon ions and GANT61 (except *SNAIL* at 2 Gy after X-ray and GANT61 exposure and *MMP9* at 0.5 Gy after carbon ion and GANT61 exposure). This decrease could explain the decreased migration we observed for DAOY cells after the addition of GANT61 when compared to the DMSO treated controls for both X-rays and carbon ions. Unfortunately, we were not able to obtain the migration samples for the proton and GANT61 combination in this study. It would however be interesting to gather this data since medulloblastoma is a good candidate for proton therapy and, metastasis is often observed in these patients.

In conclusion, we show that carbon ions are more effective in decreasing cell survival, decreasing migration, and inducing more significant alterations in the Hh pathway genes in PC3 and DAOY cells as compared to X-rays and protons. In addition, we show here for the first time that the Hh inhibitor GANT61 is able to sensitize DAOY medulloblastoma cells to particle radiation (proton and carbon ion) but not to conventional X-rays. This is an important finding since it suggests that the results that were previously obtained from combination treatment strategies with X-ray therapy cannot be automatically extrapolated to particle therapy and should be investigated separately. However, our experiments have to be expanded to other cell lines of different tumor types to understand whether our findings are also relevant for other tumor types or not.

## Author Contributions

KK performed experiments at SCK•CEN, GANIL, and iThemba LABS. MM and SI designed the experimental set-up. NB, BB, and MV helped with experiments performed at GANIL. MM and RV helped with experiments performed at GANIL and iThemba LABS. CV helped with experiments performed at iThemba LABS. AJ helped with qPCR experiments. SB and KH contributed to the design of the work, as well as with interpretation of obtained data. All co-authors critically reviewed and approved the final version to be submitted to this Journal.

### Conflict of Interest Statement

The authors declare that the research was conducted in the absence of any commercial or financial relationships that could be construed as a potential conflict of interest.
